# Dual Antiplatelet Therapy before Coronary Artery Bypass Grafting; a Systematic Review and Meta-Analysis

**Published:** 2020-05-31

**Authors:** Roxana Sadeghi, Asrin Babahajian, Arash Sarveazad, Naser Kachoueian, Mansour Bahardoust

**Affiliations:** 1 Department of cardiovascular Medicine, School of Medicine, Shahid Beheshti University of Medical Sciences, Tehran, Iran.; 2 Cardiovascular Research Center, Shahid Beheshti University of Medical Sciences, Tehran, Iran.; 3 Liver and Digestive Research Center, Research Institute for Health Development, Kurdistan University of Medical Sciences, Sanandaj, Iran.; 4 Colorectal Research Center, Iran University of Medical Sciences, Tehran, Iran.; 5 Nursing Care Research center, Iran University of Medical Sciences, Tehran, Iran.; 6 Department of Cardiac Surgery, School of Medicine, Shahid Beheshti University of Medical Sciences, Tehran, Iran.; 7 Department of Epidemiology, School of Public Health, Iran University of Medical Sciences, Tehran, Iran.

**Keywords:** Dual anti-platelet therapy, coronary artery bypass, acute coronary syndrome, aspirin, clopidogrel

## Abstract

**Introduction:**

Currently, the basis of acute coronary syndrome (ACS) therapy is dual antiplatelet therapy (DAPT) with Aspirin as a nonsteroidal anti-inflammatory drug and clopidogrel as adenosine diphosphate receptor antagonists. Therefore, the aim of the present systematic review is to answer that should DAPT with Aspirin and clopidogrel be continued until coronary artery bypass grafting (CABG) in patients who have ACS?

**Methods:**

The search for relevant studies in the present meta-analysis is based on three approaches: A) systematic searches in electronic databases, B) manual searches in Google and Google Scholar, and C) screening of bibliography of related original and review articles. The endpoints included mortality rate, myocardial infarction (MI), cerebrovascular accident (CVA), reoperation, re-exploration, other cardiac events, renal failure, length of ICU and hospital stay, chest tube drainage and blood product transfusion after CABG.

**Results:**

After the initial screening, 41 articles were studied in detail, and finally the data of 15 studies were included in the meta-analysis. DAPT before CABG in patients with ACS does not increase the rate of mortality, CVA, renal failure, MI, and other cardiac events, but increases reoperation, re-exploration, length of ICU, and hospital stay. Chest tube drainage and blood product transfusion rate significantly increased in the DAPT group compared to the control group (non-antiplatelet or Aspirin alone). Increase in chest tube drainage and blood product transfusion rate indicates an increase in bleeding, so increase in reoperation, re-exploration to control bleeding, and, subsequently, increase in the length of ICU and hospital stay are expected.

**Conclusions:**

DAPT with Aspirin and clopidogrel before CABG in patients with ACS does not increase the rate of mortality, CVA, renal failure, MI, and other cardiac events despite more bleedings, and it may be suggested before CABG for better graft patency.

## Introduction

Antiplatelet drugs, due to their key role in the prevention of clot formation in the vessels, are the main treatment strategy for disorders such as ischemic stroke, angina, non-ST, and ST-elevation myocardial infarction (1). Currently, the basis of acute coronary syndrome (ACS) therapy is dual antiplatelet therapy (DAPT) with Aspirin as a nonsteroidal anti-inflammatory drug and clopidogrel as an adenosine diphosphate receptor antagonist, which reduce thrombotic and ischemic disorders (2-4). The use of DAPT in patients with ACS who are candidates for coronary artery bypass grafting (CABG) is like a double-edged sword, which on the one hand reduces the risk of ischemia but on the other hand, increases the risk of bleeding. So, the risk of bleeding following the Aspirin or clopidogrel intake alone increases by up to 20%, and after combination therapy, it increases by up to 50% (5, 6). The amount of blood lost after CABG is very important and several studies have reported evidence against antiplatelet therapy, which increases the rate of blood loss after CABG (7-10). However, antiplatelet therapy reduces the risk of ischemia and so there is strong evidence to administrate it for CABG candidates (7, 11). The decision on whether DAPT (Aspirin and clopidogrel) should be continued or discontinued at a specific time before surgery of patients undergoing CABG is crucial for optimum management of these patients. The level of evidence in existing guidelines is moderate to low and they are derived from small studies (12-14). Therefore, the available evidence on the application of DAPT in CABG patients is not enough to make a definitive decision. For achieving a definitive conclusion on this issue, performing a systematic review can be helpful. Therefore, the aim of the present systematic review is to answer that should DAPT with Aspirin and clopidogrel be continued until CABG in patients who have ACS? We assessed the effect of DAPT on mortality rate, myocardial infarction (MI), cerebrovascular accident (CVA), reoperation, re-exploration, other cardiac events, renal failure, length of ICU and hospital stay, chest tube drainage, and blood product transfusion after CABG.

## Material and Methods

**Study design**

In the present meta-analysis, data from studies that assayed DAPT with aspirin and clopidogrel before CABG were entered. PICO definition is presented as follows:

P: CABG candidates

I: DAPT with Aspirin and clopidogrel 

C: Comparison with the control group (Aspirin alone or non-antiplatelet therapy)

O: Mortality rate, MI, CVA, reoperation, re-exploration, other cardiac events, renal failure, length of ICU and hospital stay, chest tube drainage, and need for blood product transfusion after CABG

**Search strategy**

The search for relevant studies in the present meta-analysis was based on three approaches: A) systematic searches in electronic databases, B) manual searches in Google and Google Scholar, and web pages of reliable organizations (gray literature), and C) screening of bibliography of related original and review articles. Initially, keywords were selected by consulting with an expert, using MeSH and Emtree, and screening of related articles and journals. Then, searches were performed in Medline, Embase, Scopus, and Web of Science databases, separately. The search query in the Medline database is presented in an appendix. 

**Eligibility criteria**

Clinical trials, quasi-experimental and controlled studies that evaluated DAPT with Aspirin and clopidogrel before CABG surgery without applying any limitation about time, language, age, sex, and ethnicity were included in the present meta-analysis. Studies that used Aspirin or clopidogrel alone (without dual antiplatelet) as the treatment group, studies without a control group (whether non-antiplatelet therapy or Aspirin-treated alone), and retracted articles were excluded.

**Data extraction and risk of bias assessment**

The search records were imported into EndNote software and duplicated studies were removed. Two independent reviewers screened the title and abstracts of search results and after careful assessment of the studies, data were extracted and summarized in the data extraction form. In case of disagreement between the two reviewers, the third reviewer determined the result after discussions with the other two reviewers, and the relevant data was imported. The extracted data included first author’s name, publication year, country, study type, age, sample size, the interval between discontinuation and surgery (days), outcomes. If the data could not be extracted from the study, the researcher asked the corresponding author to provide the data. In the cases that outcomes and values were published at different time points, the last evaluation time was considered. If a study reported the results as a graph, the data were extracted by "data extraction from graph method", explained by Sistrom and Mergo (15). The risk of bias assessment of clinical trials was evaluated with Cochrane's proposed guideline (16). The risk of bias assessment of cohort studies was performed using the National Institute of Health (NIH) Quality Assessment Tool for Observational Cohort and Cross-Sectional Studies (17).

**Outcomes**

Assessed outcomes included mortality rate, myocardial infarction (MI), cerebrovascular accident (CVA), reoperation, re-exploration, other cardiac events, renal failure, length of ICU and hospital stay, chest tube drainage, and blood product transfusion after CABG.

**Statistics**

STATA version 14.0 (Stata Corporation, College Station, TX) was used for statistical analyses. Using "metan" command, we performed a pooled analysis (random or fixed effect analysis based on heterogeneity among studies). Findings were presented as an overall odds ratio (OR) with 95% confidence interval (95% CI). There were two separate control groups among eligible studies: non-antiplatelet therapy and Aspirin-treated alone. Therefore, we stratified the analysis in two sections according to the control groups. Heterogeneity between studies was assessed using the I2 test and p values less than 0.1 were considered as heterogeneity. Funnel Plot and Egger's tests were used to identify publication bias (18). 

## Results

**Characteristics**

**Figure 1:**
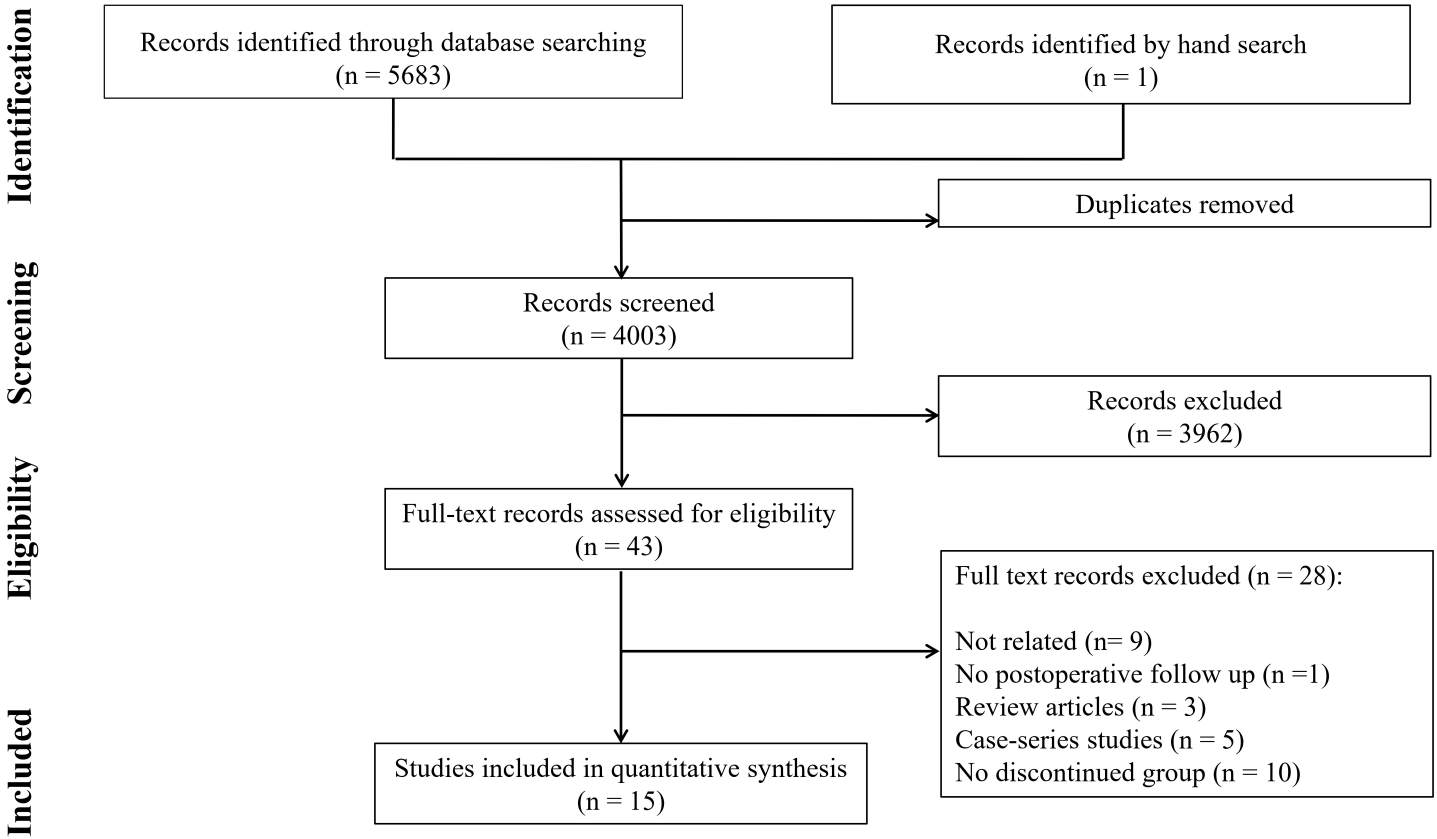
PRISMA flow diagram of the present study.

The search yielded 4003 non-duplicate records. After the initial screening, 41 studies were assessed in detail, and finally, the data of 15 studies were included in the meta-analysis (5, 6, 19-31) (Figure 1). There were 4 randomized clinical trials, 5 prospective cohort studies, and 6 retrospective cohort studies. These studies included 8029 patients in total. 6113 patients had not received any antiplatelet medication at least 5 days before CABG surgery or had only taken aspirin (non-dual APT group), and 1871 patients had used the DAPT group until the day of CABG surgery. The non-DAPT group in 7 studies had not taken any medication during the 5 days leading up to surgery, in 4 studies patients had taken Aspirin alone and the other 4 studies had two separate control arms with no-medication or Aspirin. The outcomes examined in the studies included mortality rate, MI, CVA, reoperation, re-exploration, other cardiac events, renal failure, length of ICU and hospital stay, chest tube drainage, platelet transfusion, RBC transfusion, FFP transfusion, total blood product transfusion, and major bleeding. Since major bleeding was reported in only two studies, this part of the analysis was omitted because study power was low in this section (due to the small number of entered studies). However, chest tube drainage practically indicates bleeding (Table 1).

**Risk of bias and publication bias assessment**

Four RCTs were included in the present meta-analysis. According to the Cochrane risk of bias tool (Table S1), risk of bias was low in all items in Hoxha et al. (24) study. However, in Gielen et al. (20) study, allocation concealment and blinding status were unclear. In Heidari et al. (21) study, random sequence generation had a low risk of bias and other items had unclear risk of bias. All items of Cochrane risk of bias tools had unclear/high risk of bias in Zhu et al. (31) study. 11 cohort studies were included. The risk of bias assessment based on NIH tools showed that no study reported the duration of antiplatelet therapy before allocation to groups (item 8 in Table S2). In addition, the flow of exposure-outcome assessment (item 5 in Table S2) was not reported in 7 studies, and adjustment for potential confounders was not performed in 6 studies (item 14 in Table S2). Also, the blinding status of outcome assessors and participants were not reported or were not performed in all studies (item 12 in Table S2). Publication bias assessment is presented in figures S1-S4. There is no evident publication bias in any of the analyses. 

**Comparison of DAPT versus non-antiplatelet therapy 5 days before surgery**

**Mortality**

**Figure 2:**
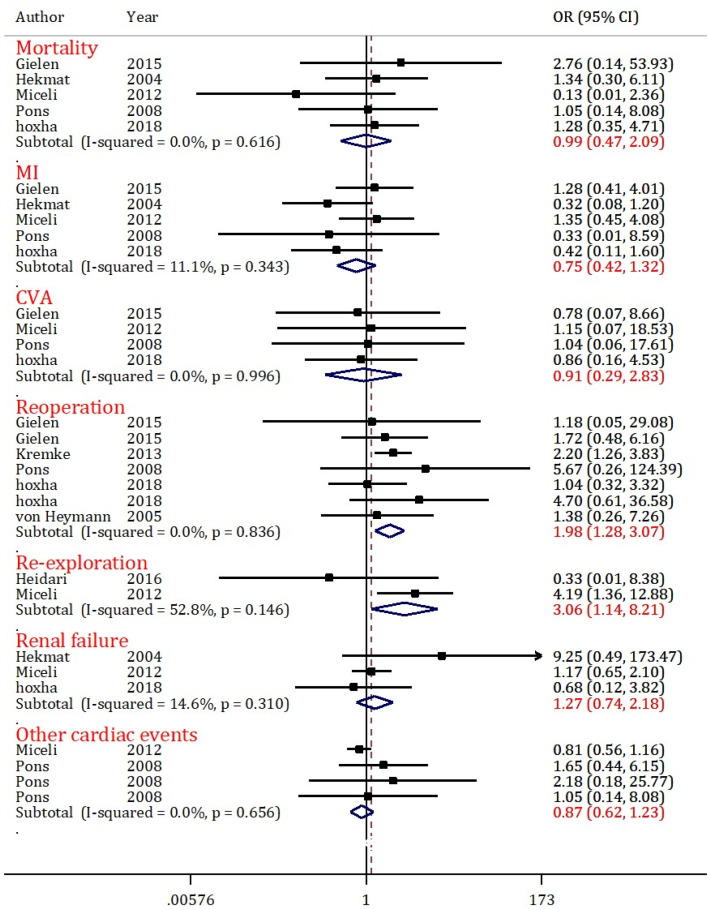
Forest plots for comparison of dual antiplatelet therapy with non-antiplatelet therapy 5 days before surgery on postoperative mortality, myocardial infarction (MI), cerebrovascular accident (CVA), reoperation and re-exploration, renal failure, and other cardiac events (atrial fibrillation, ventricular fibrillation, and heart failure) in CABG patients. CI: Confidence interval; OR: Odds ratio

Mortality data from 5 articles were included in the analyses of this section (20, 22, 24-26). Results of this section indicate heterogeneity (I2 = 0.0%; p <0.616). Figure 2 shows that the amount of mortality in the DAPT group after CABG is equal to the non-antiplatelet therapy group (OR = 2.59; 95% CI: 1.65 to 4.07).

**MI**

MI data from 5 articles were included in the analyses of this section (20, 22, 24-26). Results of this section indicate heterogeneity (I2 = 11.1%; p <0.343). Figure 2 shows that the amount of MI in the DAPT group after CABG is less than the non-antiplatelet therapy group (OR = 0.75; 95% CI: 0.42 to 1.32).

**CVA**

CVA data from 4 articles were included in the analyses of this section (20, 24-26). Results of this section indicate heterogeneity (I2 = 0.0%; p <0.996). Figure 2 shows that the amount of CVA in the DAPT group after CABG is equal to the non-antiplatelet therapy group (OR = 0.91; 95% CI: 0.29 to 2.82).

**Reoperation**

Reoperation data from 5 articles (2 articles containing two sets of data) were included in the analyses of this section (5, 20, 24, 25, 30, 32). Results of this section indicate heterogeneity (I2 = 0.0%; p <0.836). Figure 2 shows that the amount of reoperation in the DAPT group after CABG is 2 times higher than that of the non-antiplatelet therapy group (OR = 1.98; 95% CI: 1.28 to 3.07).

**Re-exploration**

Re-exploration data from 2 articles were included in the analyses of this section (21, 26). Results of this section indicate heterogeneity (I2 = 52.8%; p <0.146). Figure 2 shows that the amount of re-exploration in the DAPT group after CABG is 3 times higher than that of the non-antiplatelet therapy group (OR = 3.06; 95% CI: 1.14 to 8.21).

**Renal failure**

Renal failure data from 3 articles were included in the analyses of this section (22, 24, 26). Results of this section indicates heterogeneity (I2 = 14.6%; p <0.310). Figure 2 shows that the amount of renal failure in the DAPT group after CABG is equal to the non-antiplatelet therapy group (OR = 1.27; 95% CI: 0.74 to 2.18).

**Other cardiac events**

Other cardiac events’ data from 4 articles were included in the analyses of this section (25, 26). Results of this section indicate heterogeneity (I2 = 0.0%; p <0.656). Figure 2 shows that the amount of other cardiac events in the DAPT group after CABG is less than the non-antiplatelet therapy group (OR = 0.87; 95% CI: 0.62 to 1.23).

**Length of ICU stay**

**Figure 3:**
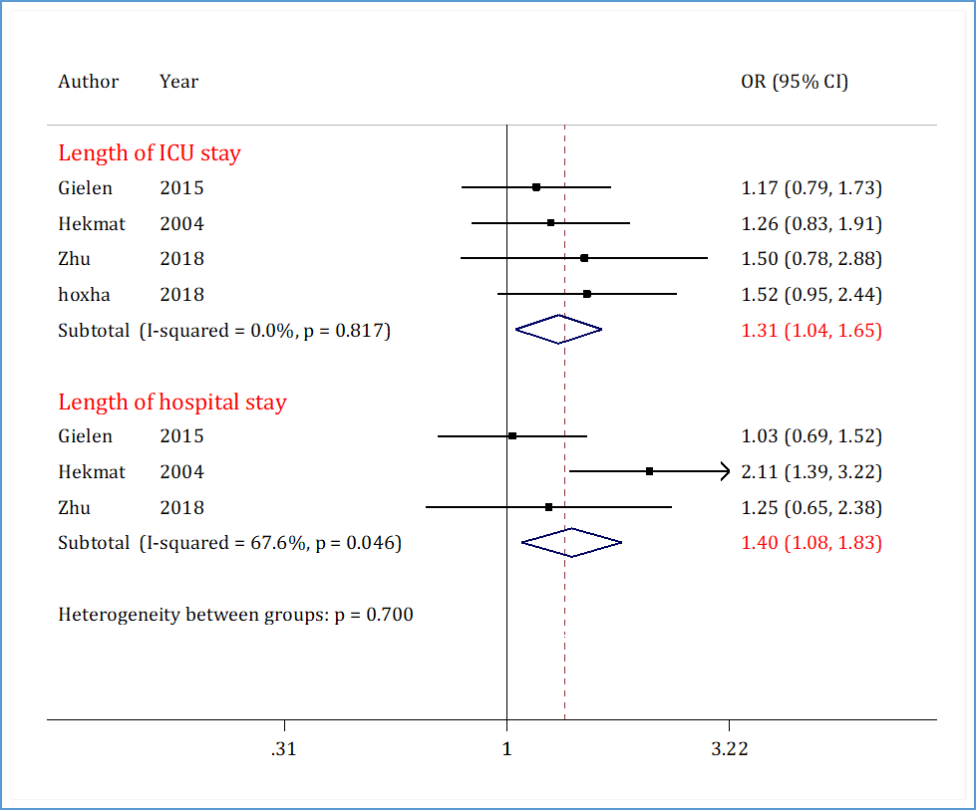
Forest plots for comparison of dual antiplatelet therapy with non-antiplatelet therapy 5 days before surgery on postoperative length of intensive care unit (ICU) stay and hospital stay in CABG patients. CI: Confidence interval; OR: Odds ratio

Length of ICU stay data from 4 articles were included in the analyses of this section (20, 22, 24, 31). Results of this section indicate heterogeneity (I2 = 0.0%; p <0.817). Figure 3 shows that the length of ICU stay in the DAPT group after CABG is 1.3 times higher than that of the non-antiplatelet therapy group (OR = 1.31; 95% CI: 1.04 to 1.65).

**Length of hospital stay**

Length of hospital stay data from 3 articles were included in the analyses of this section (20, 22, 31). Results of this section indicate heterogeneity (I2 = 67.6%; p <0.046). Figure 3 shows that the length of hospital stay in the DAPT group after CABG is 1.4 times higher than that of the non-antiplatelet therapy group (OR = 1.40; 95% CI: 1.08 to 1.83).

**Chest tube drainage**

**Figure 4:**
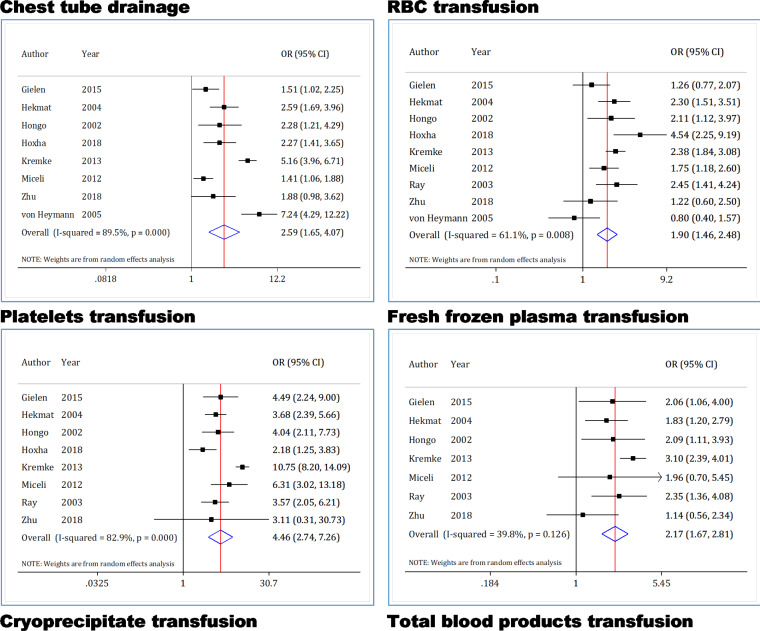
Forest plots for comparison of dual antiplatelet therapy with non-antiplatelet therapy 5 days before surgery on postoperative chest tube drainage and blood product transfusion in CABG patients. CI: Confidence interval; OR: Odds ratio; RBC: Red blood cell

Chest tube drainage data from 8 articles were included in the analyses of this section (5, 20, 22-24, 26, 30, 31). Results of this section indicate heterogeneity (I2 = 89.5%; p <0.0001). Figure 4 shows that the amount of Chest tube drainage in the DAPT group after CABG is 2.5 times higher than that of the non-antiplatelet therapy group (OR = 2.59; 95% CI: 1.65 to 4.07).

**RBC transfusion**

RBC transfusion data from 9 articles were analyzed in this section (5, 20, 22-24, 26, 29-31). Results of this section indicate heterogeneity (I2 = 61.1%; p = 0.008). Figure 4 shows that in the DAPT group, RBC transfusion value is 2 times higher than that of the non-antiplatelet therapy group (OR = 1.9; 95% CI: 1.46 to 2.48).

**Platelet transfusion**

Platelet transfusion data from 8 articles were analyzed in this section (5, 20, 22-24, 26, 29, 31). Results of this section indicate high heterogeneity (I2 = 82.9%; p <0.0001). Figure 4 shows that in the DAPT group, platelet transfusion value is 4.5 times higher than that of the non-antiplatelet therapy group (OR = 4.46; 95% CI: 2.74 to 7.26).

**Fresh frozen plasma transfusion**

Fresh frozen plasma transfusion data from 7 articles were included in the analyses of this section (5, 20, 22, 23, 26, 29, 31). Results of this section show heterogeneity (I2 = 39.8%; p = 0.126). Figure 4 shows that in the DAPT group, the amount of fresh frozen plasma transfusion after CABG is 2.2 times higher than that of the non-antiplatelet therapy group (SMD = 2.17; 95% CI: 1.67 to 2.81).

**Cryoprecipitate transfusion**

Cryoprecipitate transfusion data from 3 articles were included in the analyses of this section (23, 24, 29). Results of this section show no heterogeneity (I2 = 0.0%; p = 0.654). Figure 4 shows that in the DAPT group the amount of Cryoprecipitate transfusion after CABG did not change compared to the non-antiplatelet therapy group (OR = 1.16; 95% CI: 0.85 to 1.58).

**Total blood product transfusion**

Total blood product transfusion data from 4 articles (1 article containing two sets of data) were included in the analyses of this section (5, 24, 29, 31). Results of this section show heterogeneity (I2 = 54.8%; p = 0.065). Figure 4 shows that in the DAPT group, the amount of total blood product transfusion after CABG is 2.8 times higher than in the non-antiplatelet therapy group (OR = 2.82; 95% CI: 1.91 to 4.15).

**Comparison of DAPT versus Aspirin therapy alone until surgery**

**Mortality**

**Figure 5:**
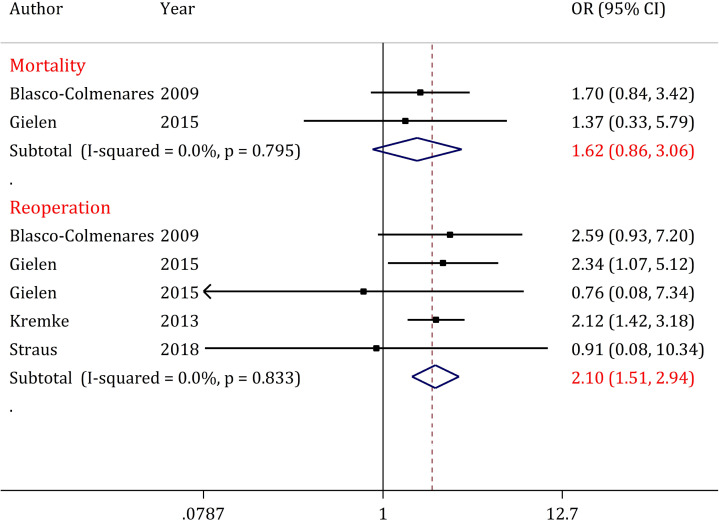
Forest plots for comparison of dual antiplatelet therapy with Aspirin therapy alone until surgery on postoperative mortality and reoperation in CABG patients. There is no evidence of publication bias. In cryoprecipitate transfusion, there are not enough studies. CI: Confidence interval; OR: Odds ratio

Mortality data from 2 articles were included in the analyses of this section (19, 20). Results of this section indicate heterogeneity (I2 = 0.0%; p <0.795). Figure 5 shows that in the DAPT group, mortality after CABG is 1.6 times higher than that of the Aspirin therapy alone group (OR = 1.62; 95% CI: 0.86 to 3.06).

**Reoperation**

Reoperation data from 4 articles (1 article containing two sets of data) were included in the analyses of this section (5, 6, 19, 20). Results of this section indicate heterogeneity (I2 = 0.0%; p <0.833). Figure 5 shows that in the DAPT group, reoperation after CABG is 2 times higher than that of the Aspirin therapy alone group (OR = 2.10; 95% CI: 1.51 to 2.94).

**Chest tube drainage**

**Figure 6:**
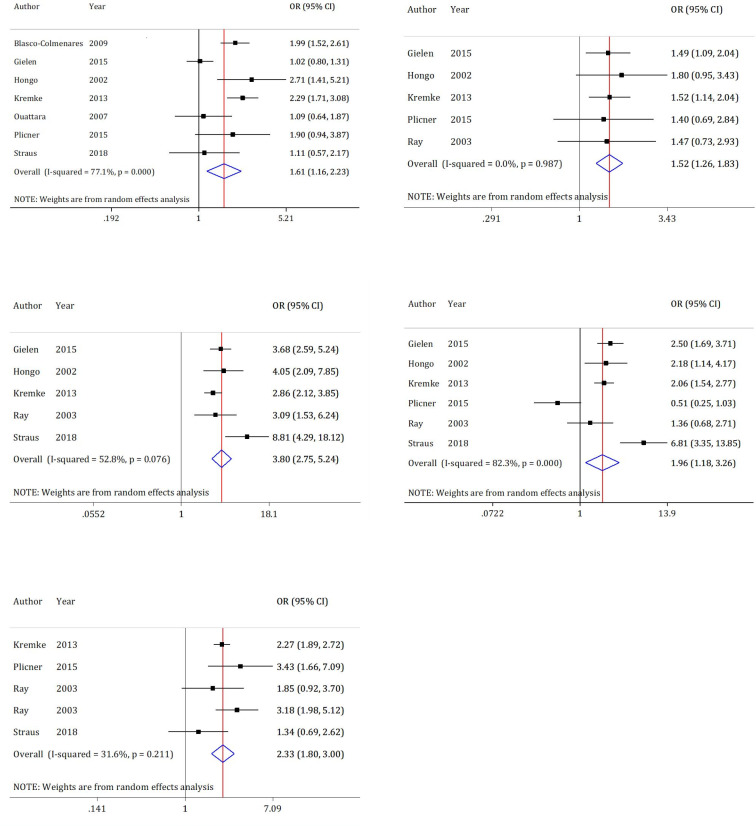
Forest plots for comparison of dual antiplatelet therapy with Aspirin therapy alone until surgery on postoperative chest tube drainage and blood product transfusion in CABG patients. There is no evidence of publication bias. In cryoprecipitate transfusion, there are not enough studies. CI: Confidence interval; OR: Odds ratio; RBC: Red blood cell; FFP: Fresh frozen plasma

Chest tube drainage data from 7 articles were included in the analyses of this section (5, 6, 19, 20, 23, 27, 28). Results of this section indicate heterogeneity (I2 = 77.1%; p <0.0001). Figure 6 shows that in the DAPT group, Chest tube drainage after CABG is 1.6 times higher than that of the Aspirin therapy alone group (OR = 1.61; 95% CI: 1.16 to 2.23).

**RBC transfusion**

RBC transfusion data from 5 articles were included in the analyses of this section (5, 20, 23, 28, 29). Results of this section show no heterogeneity (I2 = 0.0%; p = 0.987). Figure 6 shows that in the DAPT group, RBC transfusion after CABG is 1.5 times higher than that of the Aspirin therapy alone group (OR = 1.52; 95% CI: 1.26 to 1.83).

**Platelet transfusion**

Platelet transfusion data from 5 articles were analyzed in this section (5, 6, 20, 23, 29). Results of this section show heterogeneity (I2 = 52.8%; p = 0.076). Figure 6 shows that in the DAPT group, platelet transfusion after CABG is 3.8 times higher than that of the Aspirin therapy alone group (OR = 3.80; 95% CI: 2.75 to 5.24).

**Fresh frozen plasma transfusion**

Fresh frozen plasma transfusion data from 6 articles were included in the analyses of this section (5, 6, 20, 23, 28, 29). Results of this section indicate heterogeneity (I2 = 82.3%; p = 0.000). Figure 6 shows that in the DAPT group, the amount of fresh frozen plasma transfusion after CABG was 2 times higher than that of the Aspirin therapy alone group (OR = 1.96; 95% CI: 1.18 to 3.26).

**Total blood product transfusion**

Total blood product transfusion data from 4 articles (1 article containing two sets of data) were analyzed in this section (5, 6, 28, 29). Results of this section show heterogeneity (I2 = 31.6%; p = 0.211). Figure 6 shows that in the DAPT group, total blood product transfusion after CABG was 2.3 times higher than that of the Aspirin therapy alone group (OR = 2.33; 95% CI: 1.80 to 3.00).

**Table 1:** Summary of included studies

Author; year; country	Study type	Mean age	Number of females	Sample size	Number of patients in discontinued group	Number of patients in Dual APT group	APT in discontinued group	Interval between discontinuation and surgery (days)	FU	Outcome
Blasco-Colmenares; 2009; USA	RCS	66	503	1677	1483	194	Aspirin	5	24 hrs	Mortality; Reoperation; Chest tube drainage; Platelet transfusion; RBC transfusion; FFP transfusion; Total blood product transfusion;
Gielen; 2015; Netherlands	RCT	65	66	1065	775	290	Neither and aspirin	10	48 hrs	Mortality; CVA; Reoperation; MI; Length of hospital stay; Length of ICU stay; Chest tube drainage; Platelet transfusion; RBC transfusion; FFP transfusion
Heidari; 2016; Iran	RCT	58.7	31	100	50	50	Neither	5	Postoperative	Re-exploration
Hekmat; 2004; Germany	RCS	63	29	290	145	145	Neither	5	24 hrs	Mortality; MI; Renal failure; Length of hospital stay; Chest tube drainage; Platelet transfusion; RBC transfusion; FFP transfusion
Hongo; 2002; USA	PCS	66.9	60	216	165	51	Neither and aspirin	7	Postoperative	Chest tube drainage; Platelet transfusion; RBC transfusion; FFP transfusion; Cryoprecipitate transfusion
Hoxha; 2018; Albania	RCT	67.6	90	300	77	223	Neither	7	24 hrs	Mortality; MI; CVA; Renal failure; Reoperation; Length of ICU stay; Major bleeding; Chest tube drainage; Platelet transfusion; RBC transfusion; FFP transfusion; Cryoprecipitate transfusion; Total blood product transfusion
Kremke; 2013; Denmark	PCS	67	386	2205	1972	233	Neither and aspirin	5	Postoperative	Reoperation; Chest tube drainage; Platelet transfusion; RBC transfusion; FFP transfusion; Total blood product transfusion
Miceli; 2012; UK	RCS	65.2	119	618	331	287	Neither	5	Postoperative	Mortality; MI; CVA; Renal failure; Re-exploration; Other cardiac events; Chest tube drainage; Platelet transfusion; RBC transfusion; FFP transfusion
Ouattara; 2007; France	PCS	65.5	51	217	157	60	Aspirin	NA	24 hrs	Chest tube drainage
Plicner; 2015; Poland	PCS	70	26	102	50	52	Aspirin	NA	12 hrs	Chest tube drainage; RBC Transfusion; FFP Transfusion; Total blood product transfusion
Pons; 2008; Mexico	PCS	NR	36	49	25	24	Neither	6	24 hrs	Mortality; MI; CVA; Reoperation; Other cardiac events
Ray; 2003; Canada	RCS	63.3	119	648	602	46	Neither and Aspirin	7	48 hrs	Chest tube drainage; Platelet transfusion; RBC transfusion; FFP transfusion; Cryoprecipitate transfusion; Total blood product transfusion
Straus; 2018; Bosnia	RCS	62.1	35	131	41	90	Aspirin	NA	48 hrs	Reoperation; Chest tube drainage; Platelet transfusion; Total blood product transfusion
Zhu; 2018; China	RCT	48.5	50	120	60	60	Neither	7	Postoperative	Length of ICU stay; Length of hospital stay; Major bleeding; Chest tube drainage; Platelet transfusion; RBC transfusion; FFP transfusion; Total blood product transfusion
von Heymann; 2005; Germany	RCS	66.5	63	291	225	66	Neither	7	Postoperative	Reoperation; Chest tube drainage; RBC transfusion; Total blood product transfusion

APT: Antiplatelet therapy; FFP: Fresh frozen plasma; FU: Follow-up duration; PCS: Prospective cohort study; RCS: Retrospective cohort study; RBC: Red blood cells; RCT: Randomized clinical trial; MI: myocardial infarction; CVA: cardiovascular accident; ICU: intensive care unit.

## Discussion

The aim of the present systematic review and meta-analysis is to answer the question that should DAPT with Aspirin and clopidogrel or be discontinued before CABG in patients with ACS or not? To answer this question, in this systematic review, rate of mortality, MI, CVA, reoperation, re-exploration, other cardiac events (atrial fibrillation, ventricular fibrillation, and heart failure), renal failure, length of ICU and hospital stay, chest tube drainage, and blood product transfusion (RBC, Platelets, FFP, Cryoprecipitate and total blood products) were assessed after CABG. Findings showed that DAPT before CABG in patients with ACS does not increase the rate of mortality, CVA, renal failure, MI, and other cardiac events, but increases the reoperation rate, need for re-exploration, length of ICU and length of hospital stay. Chest tube drainage and blood product transfusion rate in the DAPT group had significantly increased compared to the control group (non-antiplatelet or Aspirin alone). Increased chest tube drainage and blood product transfusion rate is the result of an increased risk of bleeding following antiplatelet therapy. Bleeding also increases the need for reoperation, re-exploration, and subsequently prolongs the length of ICU and hospital stay. 

There was only one study on the assessment of atrial fibrillation, ventricular fibrillation, and heart failure following DAPT in patients with ACS; therefore, it was not possible to analyze them conclusively. Finally, all of them were reported as other cardiac events.

Since DAPT significantly increased the rate of chest tube drainage and blood product transfusion compared to both control groups (without APT and Aspirin alone), it seems that the effect of clopidogrel on bleeding and blood transfusion is prominent. In a systematic review and meta-analysis published by Cao et al., in 2014, the question was raised whether clopidogrel should be discontinued before CABG surgery? In their study, 2632 patients with ACS were studied, 1759 of them had discontinued clopidogrel less than 5 days before CABG, and 873 of them had discontinued clopidogrel more than 5 days before CABG. After comparing the results of these two groups, Cao et al. reported that patients who discontinued clopidogrel more than 5 days before CABG had significantly less major bleeding incidents (33). A systematic review and meta-analysis performed by Nijjer et al., in 2011, sought to answer the question whether clopidogrel consumption until CABG day is safe for patients with ACS? The findings of their study suggest that APT up to CABG day increases chest tube drainage and blood product transfusion (RBC, Platelets, and FFP) (34). The findings of our study are also in agreement with the results of the study by Nijjer et al. In their study, they pointed to the prominent role of heterogeneity in interpreting findings. They stated that despite increases in chest tube drainage and blood product transfusion, there was high heterogeneity in these outcomes (I2 is between 94 and 99%). Therefore, to find the origin of this heterogeneity, they divided the studies into three categories according to the year of surgery, 1999-2002, 2003-2006 and 2007 onwards. By analyzing these three groups, they found that recently published studies (2007 onwards) indicate that blood product transfusion and chest tube drainage are not different between the APT and non-APT groups, and this has been related to the development of surgical skill and technique in recent years. They eventually stated that the existence of such high heterogeneity is a serious obstacle to a definitive conclusion. In our study, moderate heterogeneity is presented in the results, which is lower than that of Nijjer et al. study. Also, there is no heterogeneity in FFP transfusion, cryoprecipitate transfusion, RBC transfusion, and total blood product transfusion results. Therefore, our results are more reliable. Another point that Nijjer and colleagues noted, was the lack of blinding in studies, that could influence surgery and its outcomes. In our study, the quality control of the studies indicated that blinding had not been applied, which can affect the conclusion.

## Limitation

In the present meta-analysis, when comparing the DAPT group with the control group, using aspirin alone (not any anticoagulants), the number of articles was so small that only two outcomes of mortality and reoperation could be statistically assessed. There were two articles about mortality, so although the results of the meta-analysis show that the chances of mortality have not increased (compared to when the control group includes aspirin alone), the conclusion should be made with caution. The present study included 15 articles, only 4 of which were clinical trials and 11 were cohort studies. Since the level of evidence presented in cohort studies is lower than clinical trials, the level of evidence presented in the present study is moderate. In addition, only two studies had reported major bleeding, thus it was not possible to present results in this regard. Another limitation of the present study is the lack of blinding in most studies, indicating the presence of probable observer bias in the studies.

## Conclusion

Our findings show that although DAPT with Aspirin and clopidogrel before CABG in patients with ACS does not increase the rate of mortality, CVA, renal failure, MI, and other cardiac events, given the significant increase in chest tube drainage, blood product transfusion, reoperation, re-exploration, and ICU and hospital stay, it is concluded that DAPT should be discontinued. However, most of the included studies were retrospective/prospective cohorts and further clinical trials are needed to reach a definitive conclusion. 
